# Effect of natural and commercially produced juices on colour stability of microhybrid and nanohybrid composites

**DOI:** 10.1038/s41405-022-00102-y

**Published:** 2022-04-20

**Authors:** Razieh Meshki, Marjan Rashidi

**Affiliations:** School of Dental Medicine, Ahvaz Jundishapur University of Medical Sciences, Ahvaz, Iran

**Keywords:** Dentistry, Paediatric dentistry

## Abstract

**Objective:**

This study aims to evaluate the discoloration rate of two types of composites, microhybrid and nanohybrid, after exposure to natural and commercially-produced juices.

**Materials and methods:**

In this experimental study, 90 disc-shaped specimens with a thickness of 2 mm and a diameter of 10 mm were taken from two composites, microhybrid P_4_ (Kerr-ITALY) and nanohybrid Filtek Z250XT (3M-ESPE-USA) (two groups of 45). Then, the samples of each group were divided into five subgroups of nine and were immersed for 10 days for 4 h in five solutions of commercially-produced orange juice, natural orange juice, commercially-produced pomegranate juice, natural pomegranate juice, and distilled water (control group). The colour of the samples was measured by a reflective spectrophotometer using the CLEl*a*b colour space at baseline and after discoloration.

**Result:**

The independent *t*-test showed that the mean discoloration rate (∆*E*) of nanohybrid composite exposed to commercially-produced orange juice, natural pomegranate juice, and commercially-produced pomegranate juice was significantly higher than microhybrid composite (*P* < 0.01). In addition, the test found that the highest discoloration rate of the nanohybrid composite was related to the effect of commercially-produced orange juice (∆*E* = 13.03) and the highest discoloration rate of microhybrid composite was related to the effect of natural pomegranate juice (∆*E* = 4.79).

**Conclusion:**

According to the results, it seems that microhybrid composites are more resistant to discoloration than nanohybrid composites. According to the results, consumption of dyed drinks, particularly natural pomegranate juice, commercially-produced orange juice, and commercially-produced pomegranate juice, is not recommended in the first few days after composite restoration.

## Introduction

Various methods have been proposed for classifying composites, one of which is based on the size and shape of filler particles and how they are distributed [1–3]. Accordingly, three groups of composites that are more widely used today include microphilic composites, microhybrids, and nanocomposites [1, 4]. Nanofield composites have recently been developed. In these materials, nanofiller particles have been used to further improve optical and physical properties of resins [5–7]. The strength and beauty of nanocomposites allow the clinician to use them to repair anterior and posterior teeth [5, 8]. The success of dental restorations depends on compressive strength, tensile strength, flexibility, and wear and tear resistance [9]. In addition, the aesthetic considerations of restorative materials require that they restore the natural appearance of the tooth, which is directly related to colour matching and colour stability. However, when restorative composites are exposed to the oral environment, they tend to change colour [9, 10].

Despite the great advances in the construction of dental composites, their colour stability is still a major problem. Resin discoloration occurs due to inherent and external factors. Internal factors such as composite resin matrix and incomplete polymerisation have a significant effect on colour stability, which is usually related to chemical degeneration of filler-resin bond and solubility of the resin matrix [6, 8, 9]. In light cure composite, camphorquinone is usually used as an optical primer to harden the composite. However, if the curing process is insufficient, discoloration of composite resin can occur and untransformed light primer leads to discoloration of restoration to yellow. In addition, other components of the optical primer system, namely aromatic or aliphatic amines, which act as accelerators, tend to change to composite restorations to yellow or brown under the influence of light or heat [5, 6].

On the other hand, external factors such as absorption of external stains are still a major problem in cosmetic repairs. The discoloration rate is affected by a number of factors, including water absorption, chemical reaction, eating habits, smoking, poor oral hygiene, and smooth surface restoration [6, 8, 9]. Consumption of certain drinks may also affect the aesthetic and physical properties of composite resins and thus impair the quality of restoration. The chemicals in beverages can weaken and degrade the surfaces of composite restorations and lead to ugly external pigmentation [11]. Previous studies on colour stability have shown that beverages such as coffee, tea, cola, and mouthwashes have varying degrees of staining on light-cure and self-cure composite resin restorations. The staining potential of these beverages and solutions is based on their composition and properties [5, 12]. Discoloration can be assessed visually or through instrumental techniques. Instrumental techniques eliminate human bias when comparing visual colour. Therefore, spectrophotometers and colorimeters are widely used to detect discoloration in dental restorations [8, 13]. Given that juices are commonly consumed, this experiment aims to determine the effect of natural and commercially-produced juices on the colour stability of microhybrid and nanohybrid composites.

## Materials and methods

### Population, sampling and design

This is an experimental laboratory study. The studied population included two groups of nanohybrid and microhybrid composites. According to the required statistical analysis, the sample size was set at 45 for each composite. In order to determine the sample size, mean comparison formula was used, where *α* = 0.1 and *β* = 0.1; according to literature, $\bar X_1 = 1.712$; $\bar X_2 = 1.065$; *S*_1_ = 1.175, *S*_2_ = 0.5 (4); thus, the minimum sample size was set at 43. To ensure the accuracy of the sample size for each composite, it was set at 45.

$$n = \frac{{(Z_{1 - \alpha /2} + Z_{1 - \beta })^2(S_1^2 + S_2^2)}}{{(\overline X _1 - \overline X _2)^2}}$$

$$n = \frac{{(1/96 + 1/28)^2(1/175^2 + 0/5^2)}}{{(1/712 - 1/065)^2}} = 43$$

### Procedure

Two composite, P_4_ microhybrid (Kerr-ITALY) and Filtek Z250XT nanohybrid (3M-ESPE-USA) shade A2 was used; 45 disc-shaped specimens of each composite were taken by cylindrical generators with a thickness of 2 mm and a diameter of 10 mm similarly in a standard way. When taking the specimens, two glass slabs below and above the generator were used to create a smooth surface and prevent the formation of non-polymerised layers. After placing the composite parts inside the generator to prevent the formation of a composite bubble, it was packed by a condenser. Once the generator was full, another glass slab was placed on it and a 5 kg weight was placed on it for 3 min to ensure the complete removal of bubbles and uniformity of the specimen. The specimens were then cured for 60 s on both sides (120 s in total) by a 550 mW/cm^2^ light cure device (Bonart Co Ltd). In the next step, the specimens were polished by silicon carbide paper discs (soflex-3M-ESPE-ultra-thin, USA) to minimise discoloration due to the surface roughness of the composites. The final thickness of the discs was 2 mm and all areas were measured with a caliper. In the next step, all specimens were kept in distilled water for 48 h for primary water absorption and completion of the polymerisation process and proximity to oral conditions. Specimens of each type of composite were randomly divided into five subgroups of nine.

Group 1: 9 microhybrid composites immersed in natural orange juice.

Group 2: 9 microhybrid composites immersed in commercially-produced orange juice (Sunich, Iran).

Group 3: 9 microhybrid composites immersed in natural pomegranate juice.

Group 4: 9 microhybrid composites immersed in commercially-produced pomegranate juice (Sunich, Iran).

Group 5: 9 nanohybrid composites immersed in natural orange juice.

Group 6: 9 nanohybrid composites immersed in commercially-produced orange juice (Sunich, Iran).

Group 7: 9 nanohybrid composites immersed in natural pomegranate juice.

Group 8: 9 nanohybrid composites immersed in commercially-produced pomegranate juice (Sunich, Iran).

The eight subgroups were immersed for 10 days, 4 h a day (40 h in total) in the juices, and 20 h in distilled water at 37 °C. The fifth subgroup of each type of composite, as a control group, was immersed in distilled water at 37 °C for 10 days [1]. pH of beverages was measured before immersing. Beverages were consumed at a normal temperature of about 4 °C. Beverages were also replaced daily to prevent possible interactions. In addition, the specimens were cleaned with distilled water and a soft toothbrush for 30 s each time after removing them from the solution. At the end of the 10th day, the specimens were transferred to a reflective spectrophotometer to measure the colour after the discoloration stage.

### Data analysis

In order to analyse the data, descriptive statistical methods including frequency, mean, standard deviation, and tables and graphs were used. In order to examine the hypotheses by examining the assumptions of parametric statistics such as Kolmogorov Smirnov test (assumption of normality) of Levine test (assumption of equal variances), independent *t*-tests were used; in order to compare means, LSD follow-up test was used. The data were analysed by SPSS20 software.

## Results

In this study, 45 specimens of each of the Filtek Z250XT nanohybrid (3M, ESME, USA) and P_4_ microhybrid (Kerr, ITALY) composites were taken and three parameters of L* (brightness), a* (red-green), and b* (blue-yellow) were used as initial records. On the tenth day, they were measured and recorded using a spectrophotometer. The overall discoloration of the specimens was calculated by the following formula:

$${\Delta}E^{\ast} = [(L_1^ \ast - L_0^ \ast )^2 + (a_1^ \ast - a_0^ \ast )^2 + (b_1^ \ast - b_0^ \ast )^{1/2}]$$

The collected data were statistically analysed. The findings are as follows:

### Determining and comparing discoloration of microhybrid composite exposed to natural and commercially-produced juices

Table 1 reports the mean and standard deviation of discoloration of microhybrid composite exposed to natural and commercially-produced juices and the results of an independent *t*-test to compare the effect of natural and commercially-produced juices on the discoloration of microhybrid composite.

**Table 1:** Comparing discoloration rate of microhybrid composite exposed to natural and commercially-produced juices.

Variable	Beverage	Natural Mean ± SD	Commercially-produced Mean ± SD	*P* value
Discoloration rate of microhybrid composite	Pomegranate juice	4.79 ± 0.2.72	3.10 ± 0.61	0.088
	Orange juice	2.16 ± 0.88	3.02 ± 0.61	0.447

*SD* standard deviation.

As shown in Table 1, the mean discoloration of microhybrid composite exposed to natural pomegranate juice is 4.79, exposed to commercially-produced pomegranate juice is 3.10, exposed to industrial orange juice is 3.02, and exposed to natural orange juice is 2.16. The results of the independent *t*-test showed no significant difference between the mean discoloration of microhybrid composite in the two groups of natural and commercially-produced juices (*P* > 0.05).

**Fig. 1:**
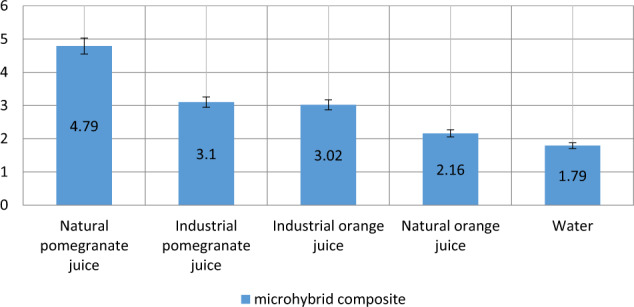
Mean discoloration of microhybrid composites exposed to different beverages.

As shown in Fig. 1, the highest discoloration of the microhybrid composite was related to the effect of natural pomegranate juice (mean 4.79), followed by commercially-produced pomegranate juice (3.1), commercially-produced orange juice (3.02), natural orange juice (2.16) and water (1.79).

### Determining and comparing discoloration of nanohybrid composite exposed to natural and commercially-produced juices

Table 2 reports the mean and standard deviation of discoloration of nanohybrid composite exposed to natural and commercially-produced juices and the results of an independent *t*-test to compare the effect of natural and commercially-produced juices on the discoloration of microhybrid composite.

**Table 2:** Comparing discoloration rate of nanohybrid composite exposed to natural and commercially-produced juices.

Variable	Beverage	Natural Mean ± SD	Commercially-produced Mean ± SD	*P* value
Discoloration rate of nanohybrid composite	Pomegranate juice	8.54 ± 2.20	4.66 ± 1.51	0.001*
	Orange juice	3.14 ± 1.29	13.03 ± 8.90	0.010*

*SD* standard deviation; the difference is statistically significant

*P < 0.01

As shown in Table 2, the mean discoloration of nanohybrid composite exposed to natural pomegranate juice is 8.54, exposed to commercially-produced pomegranate juice is 4.66, exposed to natural orange juice is 3.14, and exposed to industrial orange juice is 13.03. The results of the independent *t*-test showed that the mean discoloration rate of nanohybrid composite exposed to natural pomegranate juice was significantly higher than commercially-produced pomegranate juice (*P* = 0.001), while the mean discoloration rate of nanohybrid composite exposed to commercially-produced orange juice was significantly higher than natural orange juice (*P* = 0.010).

**Fig. 2:**
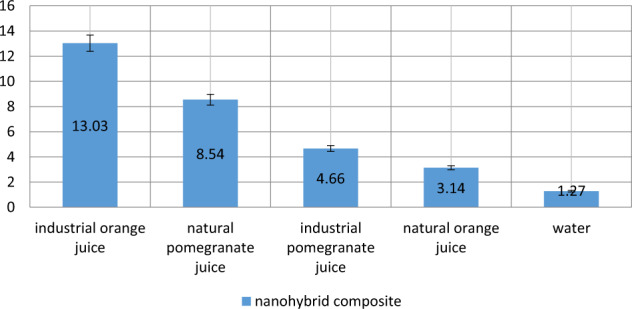
Mean discoloration of nanohybrid composites exposed to different beverages.

As shown in Fig. 2, the highest discoloration of the nanohybrid composite was related to the effect of commercially-produced orange juice (mean 13.03), followed by natural pomegranate juice (8.54), commercially-produced pomegranate juice (4.66), natural orange juice (3.14) and water (1.27).

### Determining and comparing discoloration of nanohybrid and microhybrid composites exposed to natural and commercially-produced juices

An independent *t*-test was used to compare the mean discoloration of nanohybrid and microhybrid composites exposed to natural and commercially-produced juices (Table 3).

**Table 3:** Comparing discoloration rate of microhybrid and nanohybrid composites exposed to natural and commercially-produced juices.

Variable	Beverage	Microhybrid Mean ± SD	Nanohybrid Mean ± SD	*P* value
Discoloration rate	Natural pomegranate juice	4.79 ± 2.72	8.54 ± 2.20	0.006*
	Commercially-produced pomegranate juice	3.10 ± 0.61	4.66 ± 1.51	0.004*
	Natural orange juice	2.16 ± 0.88	3.14 ± 1.29	0.079
	Commercially-produced orange juice	3.02 ± 3.16	13.03 ± 8.90	0.010*
	Water	1.79 ± 0.64	1.27 ± 0.63	0.100

*SD* standard deviation; the difference is statistically significant.

*P < 0.01.

**Fig. 3:**
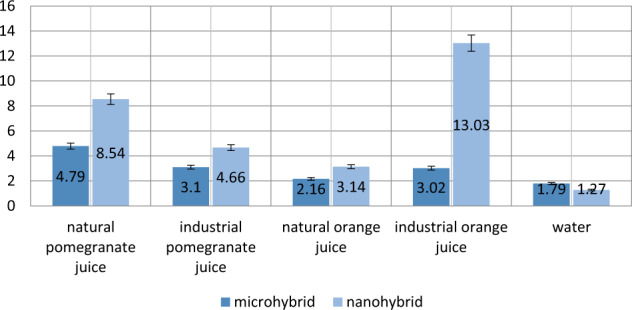
Mean discoloration of microhybrid and nanohybrid composites exposed to natural and commercially-produced juices.

As shown in Table 3, the results of the independent *t* test showed that the mean discoloration rate of nanohybrid composite exposed to natural pomegranate juice, commercially-produced pomegranate juice, and industrial orange juice was significantly higher than microhybrid composite (*P* < 0.001); no significant difference was found between mean discoloration of microhybrid and nanohybrid composites exposed to natural orange juice and water (*P* > 0.05) (Fig. 3).

## Discussion

Long-term colour stability of tooth-coloured restorative materials is important not only in terms of aesthetics but also in terms of reducing the additional costs of treatment, which is related to frequent replacement of dental restorations. Colour stability of restorations during their functional life is critical to the acceptability of restoration. Discoloration in dental composites is due to multiple reasons and depends on inherent factors such as chemical changes in materials (resin matrix filler particles) and particle–matrix boundaries, and external factors such as adsorption or absorption of stains, diet, smoking habits, and water absorption of resin monomers [14]. One of the problems we may encounter when using composites is their lack of complete polymerisation due to insufficient light intensity and insufficient light exposure time. Composites that are not fully polymerised have more water absorption and solubility, which is evident in the clinic as early colour instability [3]. In this study, the polymerisation time of all specimens was sufficient and equal. When the composite surface is cured against a transparent celluloid strip and subsequently it is not finished, a resin-rich surface is formed, which due to low physical properties, the surface layer undergoes discoloration more than the finished surfaces; however, a finished surface creates a filler-rich surface with higher Knoop hardness values and less prone to chemical solubility [1, 15]. Therefore, all specimens were finished in a standard and uniform form. When the composite is adjacent to liquids, most of the water absorption occurs by organic polymer matrix in the first 4 days and the highest water absorption occurs during the first week [3]. Composite resins that can absorb water are able to absorb other liquids with pigments that lead to discoloration. It is assumed that water acts as a means of penetrating the dye into the resin matrix [16]. Because colour is a physical-psychological phenomenon that varies from person to person and even in a person at different times, measuring it with precision instruments eliminates the subjective errors of evaluation. Therefore, in this study, colour measurement was performed using a reflective spectrophotometer, the accuracy of which has been confirmed in various studies [1, 17]. In the present study, common natural and commercially-produced juices including natural and industrial orange juice, natural and commercially-produced pomegranate juice, and distilled water (control group) were used; due to the fact that the highest absorption rate occurs during the first 7–10 days, the period for determining the colour of the specimens was determined 10 days after the specimens were placed in the solutions.

The findings of this study showed that microhybrid composite in natural pomegranate juice solution had an unacceptable discoloration (∆*E* = 4.79) and nanohybrid composite in three solutions of industrial orange juice (∆*E* = 13.03), natural pomegranate juice (∆*E* = 8.54), and industrial pomegranate juice (∆*E* = 4.66) had unacceptable discoloration, which was visually noticeable. Discoloration rate of nanohybrid composite exposed to commercially-produced orange juice, natural pomegranate juice, and commercially-produced pomegranate juice was significantly higher than microhybrid composite (*P* < 0.01) and there was no significant difference between mean discoloration of microhybrid and nanohybrid composites exposed to natural orange juice and water (*P* > 0.05). In this regard, studies have examined the effect of different beverages on the colour stability of composites.

Al-Haj Ali et al. examined the effect of common soft drinks (iced tea, sports drink, orange juice, Cola, and distilled water) on the colour stability of microhybrid composites and nanocomposites. The results showed that microhybrid composite has higher colour stability in all soft drinks [18]. Kheraif et al. examined the effect of coffee, tea, cola, and distilled water on colour stability and conversion degree of nano- and microhybrid composites. The results showed that nanohybrid composites with a high conversion degree had the lowest colour stability and had a significant discoloration compared to microhybrid composites [19]. Bansal et al. examined the effect of alcoholic and non-alcoholic beverages on the colour stability of nanofield and microhybrid composites. The results showed that microhybrid composite has higher colour stability in different beverages [11]. The results of these studies are consistent with the current study and suggest that microhybrid composite has higher colour stability than nanohybrid composite.

Effective factors on the sensitivity of composites to discoloration include filler type, resin type, and staining agent. Microhybrid composite is a glass-ceramic composite with different sizes and distributions of filler particles. This composite has 77% by weight of microfillers and very fine particles whose size is about 0.05 μm. This glass structure must provide optimal stability. It has also been reported that increasing the particle size causes less discoloration due to the reduced matrix filler [9]. Nanohybrid composites, on the other hand, contain agglomerate particles called nanoclusters. These particles are less resistant to discoloration than silicon-zirconia micron-sized fillers in microhybrid composite, which can be due to their high water absorption properties [5].

It has been reported that smaller filler particles are removed during polishing and finishing operations in nanohybrids, and small voids remain at the surface of the restorative material compared to microhybrids. This advantage of nanohybrids does not seem to make them resistant to staining [5]. On the other hand, some studies contradict the results of the current study.

Kumar et al. stated that the colour stability of microhybrid composite after 24 and 48 h of exposure to red wine and cola is less than that of nanohybrid composite [20]. Reddy et al. examined the effect of cola, coffee, and tea on the colour stability of nano, microhybrid, and hybrid resin composites. The results showed nanofilled composites have less colour change than microhybrid and hybrid composite resin [21]. Erdemir et al. examined the effect of sports drinks on the colour stability of microhybrid and nanofilled composites after 1 month and 6 months. The results showed that the highest discoloration occurred during 1 month in microhybrid composite and the lowest discoloration occurred during 6 months in nanofilled composite [8]. Contrary to previous studies, some scientists believe that due to the large size and high stiffness of filler particles in microhybrid composites, the resin matrix tends to wear harder than fillers, and this clinically leads to rough surfaces after finishing, restoration wear, after long-term function and increased sensitivity to discoloration in microhybrids [20].

In both groups of microhybrid and nanohybrid composites, there was a logical relationship between pH of natural and industrial juices and discoloration of the composites, in the way that commercially-produced orange juice with pH = 3.7 and natural pomegranate juice with pH = 4 compared to natural orange juice (pH = 5.3) and commercially-produced pomegranate juice (pH = 4.8) lead to more discoloration in both groups. Bansal et al. also found a logical relationship between pH and discoloration of composites. Coca-Cola, with the lowest pH (pH = 1.57) among beverages, led to the highest discoloration in composites [11]. In the end, it is worth mentioning that the oral cavity is a complex environment in which several factors are involved. Saliva dilutes the solutions used and changes their pH; in addition, it contains various enzymes and effective salts. Composites are exposed to a wide range of thermal changes following consumption of hot and cold foods and beverages, and their physical properties change over time [1, 17].

## Conclusion

In general, the findings of this study showed that the colour stability of Filtek Z250XT nanohybrid composite (3M, ESME, USA) is significantly lower than that of P_4_ microhybrid composite (Kerr, ITALY). Considering the obtained results, consumption of industrial orange juice, natural pomegranate juice, and commercially-produced pomegranate juice is not recommended in the first few days after composite restoration. It is also suggested that future studies examine the effect of natural and commercially-produced juices on colour stability of microhybrid and nanohybrid composites clinically, the effect of natural and commercially-produced juices on other commercial brands of nanohybrid and microhybrid composites, the effect of natural and commercially-produced juices on other common types of composites used in restorative dentistry.
